# Skin and soft tissue infections in hospitalized and critically ill patients: a nationwide population-based study

**DOI:** 10.1186/1471-2334-10-151

**Published:** 2010-06-04

**Authors:** Hsiu-Nien Shen, Chin-Li Lu

**Affiliations:** 1 Department of Intensive Care Medicine, Chi Mei Medical Center, No. 901 Chung-Hwa Road, Yong-Kang City, Tainan, Taiwan; 2 Department of Medical Research, Chi Mei Medical Center, No. 901 Chung-Hwa Road, Yong-Kang City, Tainan, Taiwan

## Abstract

**Background:**

The proportional distributions of various skin and soft tissue infections (SSTIs) with/without intensive care are unclear. Among SSTI patients, the prevalence and significance of complicating factors, such as comorbidities and infections other than skin/soft tissue (non-SST infections), remain poorly understood. We conducted this population-based study to characterize hospitalized SSTI patients with/without intensive care and to identify factors associated with patient outcome.

**Methods:**

We analyzed first-episode SSTIs between January 1, 2005 and December 31, 2007 from the hospitalized claims data of a nationally representative sample of 1,000,000 people, about 5% of the population, enrolled in the Taiwan National Health Insurance program. We classified 18 groups of SSTIs into three major categories: 1) superficial; 2) deeper or healthcare-associated; and 3) gangrenous or necrotizing infections. Multivariate logistic regression models were applied to identify factors associated with intensive care unit (ICU) admission and hospital mortality.

**Results:**

Of 146,686 patients ever hospitalized during the 3-year study period, we identified 11,390 (7.7%) patients having 12,030 SSTIs. Among these SSTI patients, 1,033 (9.1%) had ICU admission and 306 (2.7%) died at hospital discharge. The most common categories of SSTIs in ICU and non-ICU patients were "deeper or healthcare-associated" (62%) and "superficial" (60%) infections, respectively. Of all SSTI patients, 45.3% had comorbidities and 31.3% had non-SST infections. In the multivariate analyses adjusting for demographics and hospital levels, the presence of several comorbid conditions was associated with ICU admission or hospital mortality, but the results were inconsistent across most common SSTIs. In the same analyses, the presence of non-SST infections was consistently associated with increased risk of ICU admission (adjusted odds ratios [OR] 3.34, 95% confidence interval [CI] 2.91-3.83) and hospital mortality (adjusted OR 5.93, 95% CI 4.57-7.71).

**Conclusions:**

The proportional distributions of various SSTIs differed between ICU and non-ICU patients. Nearly one-third of hospitalized SSTI patients had non-SST infections, and the presence of which predicted ICU admission and hospital mortality.

## Background

Skin and soft tissue infections (SSTIs) are common in outpatient clinic and emergency department visits [1-4] and include a wide variety of infections of the epidermis, dermis, subcutaneous tissue, fascia and muscle [3,4]. SSTIs usually result from traumatic, surgical or healthcare-related skin break down with secondary inflammatory microbial invasions [4]. The severity of SSTIs ranges from mild superficial to deeper or potentially fatal necrotizing infections requiring hospitalization or intensive care [2-8].

Among hospitalized or critically ill patients, several epidemiological studies have shown that about 4.3%-10.5% of septic episodes are caused by SSTIs [9-12]. But data on the proportional distributions of SSTIs with/without intensive care are still limited [5,8,13-15]. The available data suggest a marked difference in the proportional distribution between those with/without intensive care. For example, based on a National Inpatient Sample from about 20% of all United States community hospitals, Edelsberg et al suggested that most hospitalized SSTIs were either "superficial" (58.6%) or "deeper and/or healthcare-associated" (40.1%) infections; the proportion of "often fatal" SSTIs was relatively low (1.3%) [5]. In another large database study on dermatological conditions in the intensive care unit (ICU) [8], only 0.4% of all ICU admissions had SSTIs, and about 60% of which were necrotizing fasciitis, a potentially fatal infection. Another two studies, including only "superficial" and "deep and/or healthcare-associated" infections, have shown that about 2.0%-5.8% of hospitalized SSTI patients are admitted to the ICU [14,15]. The proportional distributions of various SSTIs with/without intensive care are not reported.

Because SSTIs alone may not result in hospital or ICU admission, complicating factors such as comorbidities or associated infections other than skin/soft tissue (non-SST infections) may be present in hospitalized patients with SSTIs [8,14,15]. For example, sepsis and pneumonia have been found to be the most common non-dermatological reasons for ICU admission in patients with SSTIs [8]. However, the prevalence and significance of these complicating factors in SSTI patients remain poorly understood. Therefore, based on a nationwide population-based database, we conducted this study to investigate the characteristics and distributions of hospitalized SSTI patients with/without intensive care, to compare these differences across most common SSTIs and to identify complicating factors, such as comorbidities and non-SST infections, which might be associated with ICU admission and hospital mortality.

## Methods

### Database

A compulsory and universal National Health Insurance (NHI) program has been initiated by the Taiwan government since 1995 [16]. With the exception of prison inmates, all citizens are enrolled in the program. Patients in this study were drawn from NHI Research Database (NHIRD) [16], released for research purposes by the National Health Research Institute, Taipei, Taiwan. The NHIRD, which covers nearly all (99%) inpatient and outpatient claims for its population of >22 million, is one of the largest and most comprehensive databases in the world and has been used extensively in various studies [16-19]. The NHIRD provided encrypted patient identification numbers, gender, birthday, dates of admission and discharge, medical institutions providing the services, the ICD-9-CM (*International Classification of Diseases, Ninth Revision, Clinical Modification*) codes of diagnoses (up to five) and procedures (up to five), and outcome at hospital discharge (recovered, died or transferred out).

### Study Sample

The study cohort, a randomly selected longitudinal NHIRD dataset of 1,000,000 people enrolled in 2005 [16], represented about 5% of the Taiwanese population. There were no significant differences in age and gender between the study cohort and the general population [16]. All health care claims were collected annually for ten years, which enables us to do longitudinal follow-ups of individual patients of the cohort during the study period, from 2004 to 2007 (inclusive), to rule out non-first-episode SSTI hospitalizations. We linked to diagnostic codes through the hospitalization claims data to identify patients with a discharge diagnosis of SSTIs and the corresponding order codes used for the study subjects. To ensure the inclusion of first-episode SSTI hospitalization between 2005 and 2007, patients were excluded if they had ever been hospitalized for the disease in 2004 or had subsequent hospitalizations after the first episodes. The Human Subjects Institutional Review Board Approval and informed consent were exempt for the use of encrypted administrative database.

### Patient selection and definition

The definitions of SSTIs were based on ICD-9-CM codes, modified from Edelsberg's classification [5] and included the following eighteen types: 1) acute lymphadenitis (ICD-9-CM code 683.x); 2) carbuncle/furuncle (680.x); 3) cellulitis/abscess of finger/toe (681.x); 4) impetigo (684.x); 5) amputation stump infection (997.62); 6) other cellulitis/abscess (682.x); 7) other local SSTIs (686.x); 8) anal/rectal abscess (566.x); 9) chronic ulcer of specified/unspecified sites (707.8,707.9); 10) decubitus ulcer (707.0); 11) infection due to vascular device/implant (996.62); 12) pilonidal cyst with abscess (685.x); 13) post-operation wound infection (998.5); 14) post-traumatic wound infection (958.3); 15) lower limb ulcer except decubitus (707.1); 16) gangrene (785.4); 17) necrotizing fasciitis (728.86); and 18) Fournier's gangrene (608.83). Based on different predominant pathogens and risk of mortality [5], these SSTIs were further grouped into three mutually exclusive categories: 1) superficial infections predominantly caused by *Staphylococcus aureus *or *Streptococcus pyogenes *(groups 1-7 above); 2) deeper or healthcare-associated infections more likely caused by anaerobic or gram-negative organisms (groups 8-15); and 3) gangrenous or necrotizing infections (or "often fatal infections") (groups 16-18).

Patients readmitted ≤1 day after discharge from the same or different hospitals were regarded as the same hospitalization. We defined a case as having surgical condition if procedure codes, other than tracheostomy, were present and their surgical charges were more than zero. Because it is a custom for many Taiwanese to "die at home", "in-hospital death" coded at discharge would underestimate true hospital mortality. Therefore, we presumed cases that were attrited from the NHI within 3 days of hospital discharge were in-hospital death because of the universal insurance coverage and unlikely emigration of the cases shortly after hospital discharge [18].

### Measurements

Demographic and clinical characteristics of study subjects were examined, including age, gender, hospital levels (medical centers, regional hospitals or district hospitals), prevalence of selected comorbid conditions, Charlson comorbidity index [20,21], bacterial or fungal infections (SSTIs and non-SST infections), and outcome. The Charlson comorbidity index is a weighted summary measure of clinically important concomitant diseases that has been adapted for use with ICD-9-CM coded administrative databases [20,21]. A comprehensive list of ICD-9-CM codes, which included 1,286 distinct codes for bacterial and fungal infections defined by Angus et al in an epidemiological study on severe sepsis [9], was used to identify non-SST infections.

### Statistics

Continuous variables were described as median (inter-quartile range, IQR); discrete ones as counts or percentages. Univariate and multivariate logistic regression analyses were applied to identify factors associated with ICU admission and hospital mortality for SSTIs. In case of small event number, only those with ≥50 ICU cases were selected for detailed analyses. The Goodness-of-fit of the logistic regression model was assessed by the Hosmer and Lemeshow test, and the explanatory power was reported with a Nagelkerke's pseudo-R-square. Data analysis was performed using a professional statistical package, SPSS for Windows, version 17.0. (SPSS Inc., Illinois, U.S.A.). A two-tailed p value of < 0.05 was considered significant.

## Results

### Demographics and clinical characteristics

Of 146,686 patients ever hospitalized during the 3-year study period, we identified 11,390 (7.7%) patients having 12,030 SSTIs. Among these SSTI patients, 1,033 (9.1%) had ICU admission and 306 (2.7%) died at hospital discharge. Demographics and clinical characteristics of all patients with SSTIs are shown in Table 1. Among non-SST infections, 76.5% of them were respiratory, intra-abdominal or genitourinary tract infection (Table 1).

**Table 1:** Demographics and clinical characteristics of hospitalized skin and soft tissue infections 2005-2007

Variables	No	%
**Variables**	11,390	100
**Age, yr**	58 (39-74)*	
**<18**	872	7.7
**18-64**	5,824	51.1
**≥65**	4,694	41.2
**Men**	6,924	60.8
**Women**	4,466	39.2
**Medical conditions**	8,230	72.3
**Surgical conditions**	3,160	27.7
**Hospital level**		
**Medical center**	3,293	28.9
**Regional hospital**	4,786	42.0
**District hospital**	3,310	29.1
**Charlson Comorbidity Index**	0 (0-1)*	
**0**	6,235	54.7
**1**	2,552	22.4
**2**	1,498	13.2
**≥3**	1,105	9.7
**Comorbid conditions**		
**Chronic obstructive pulmonary disease**	535	4.7
**Congestive heart failure**	355	3.1
**Cerebrovascular disease**	666	5.8
**Diabetes mellitus**	2,507	22.0
**End stage renal disease**	567	5.0
**Cirrhosis**	285	2.5
**Cancer**	788	6.9
**Infections other than skin/soft tissue^#^**	3,570	31.3
**Respiratory**	1,009	8.9
**Intra-abdominal**	884	7.8
**Genitourinary tract**	839	7.4
**Others**	1,494	13.1

* Values are expressed as median (inter-quartile range).

^# ^Patients may have more than one site of infection other than skin and soft tissue.

The proportional distributions of SSTIs by groups and categories are shown in Table 2. The three most common groups of SSTIs were "other cellulitis or abscess", "decubitus ulcer" and "post-operation wound infection"; and they accounted for 76.5% of all hospitalized cases and for 72.9% of ICU ones, respectively. In addition, "infection caused by vascular device/implant", "gangrene" and "necrotizing fasciitis" comprised another 24.3% of the ICU cases.

**Table 2:** Proportional distributions of skin and soft tissue infections by groups and categories 2005-2007 (patient no. = 10,390)*

Variables	n	%
**Superficial infections**	**6,840**	**56.9**
Acute lymphadenitis	136	1.1
Carbuncle/funruncle	224	1.9
Cellulitis/abscess of finger/toe	368	3.1
Impetigo	28	0.2
Amputation stump infection	28	0.2
Other cellulitis/abscess	5,982	49.7
Other local soft tissue infection	252	2.1
**Deeper or healthcare-associated infections**	**4,317**	**35.9**
Anal/rectal abscess	626	5.2
Chronic ulcer of specified/unspecified sites	57	0.5
Decubitus ulcer	1,324	11.0
Infection due to vascular device/implant	377	3.1
Pilonidal cyst with abscess	17	0.1
Post-operation wound infection	1,262	10.5
Post-traumatic wound infection	212	1.8
Lower limb ulcer except decubitus	498	4.1
**Gangrenous or necrotizing Infections**	**873**	**7.3**
Gangrene	506	4.2
Necrotizing fasciitis	357	3.0
Fournier's gangrene	42	0.3
**Total**	**12,030**	**100.0**

*Patients may have more than one group of skin and soft tissue infection.

**Figure 1:**
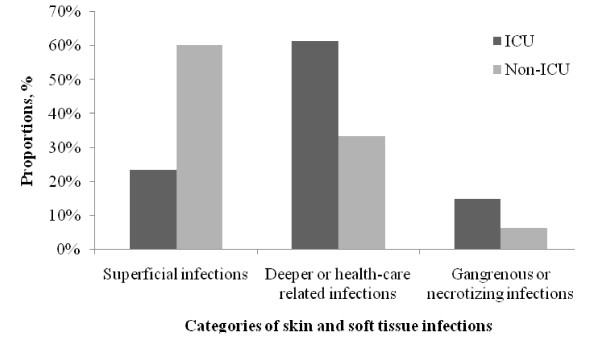
**Proportions of skin and soft tissue infections (SSTIs) in those with/without intensive care unit (ICU) admission**. (Note: Eighteen types of SSTIs are grouped into three categories according to modified Edelsberg's classification [5].)

Relative proportions of SSTIs by categories for ICU and non-ICU cases are shown in Figure 1. The most common categories of SSTIs in ICU and non-ICU patients were "deeper or healthcare-associated" and "superficial" infections, respectively (Figure 1).

Table 3 compares the patient and hospital characteristics and hospital mortality by intensive care status for the six most common SSTIs in the ICU. Among the six SSTIs, the ICU admission rates were the highest in "necrotizing fasciitis" (23.8%, 85/357), followed by "decubitus ulcer" (22.8%), "infection due to vascular device/implant" (21.2%), "post-operation wound infection" (17.8%), "gangrene" (15.4%) and "other cellulitis/abscess" (3.8%), respectively. The median ages of these patients was the highest in "decubitus ulcer" (78, IQR 69-84 yr) and the youngest in "other cellulitis or abscess" (54, 35-72 yr) and in "post-operation wound infection" (55, 25-75 yr). Diabetes mellitus was the most common comorbid condition in most SSTIs except "post-operation wound infection" and "infection due to vascular device/implant". The most common comorbid conditions in patients with "post-operation wound infection" and "infection due to vascular device/implant" were cancer (18.0%) and end stage renal disease (ESRD) (51.2%), respectively. Compared with non-ICU patients across the six most common SSTIs, ICU ones were more likely to have non-SST infections (44.9%-78.5% vs 17.8%-38.4%).

**Table 3:** Comparison of skin and soft tissue infections with and without intensive care unit (ICU) admission^$^

Variables	Other cellulitis or abscess	Decubitus ulcer	Post-operation wound infection	Infection due to vascular device/implant	Gangrene	Necrotizing fasciitis
						
	ICU (n = 227)/Non-ICU (n = 5,755)	ICU (n = 302)/Non-ICU (n = 1,022)	ICU (n = 225)/Non-ICU (n = 1,037)	ICU (n = 80)/Non-ICU (n = 297)	ICU (n = 78)/Non-ICU (n = 428)	ICU (n = 85)/Non-ICU (n = 272)
**Age ≥65 yr**	55.9/35.3*	83.1/81.8	52.0/31.6*	61.3/49.5	75.6/58.9^#^	45.9/32.4^#^
**Men**	63.0/60.7	57.3/52.2	66.7/61.6	48.8/51.2	61.5/53.5	69.4/66.2
**Surgical conditions**	37.0/10.2*	24.8/14.5*	92.9/51.3*	67.5/56.2	65.4/59.8	77.6/72.4
**Hospital levels, Center/Regional/District**	34.4, 45.4, 20.3/25.4, 42.5, 32.1*	17.2, 46.7, 36.1/20.5, 33.6, 45.9*	45.8, 44.0, 10.2/41.5, 39.7, 18.8^#^	36.3,53.8, 10.0/51.5, 42.8, 5.7	35.9, 44.9, 19.2/29.7, 39.7, 30.6	43.5, 47.1, 9.4/18.4, 62.1, 19.5*
**Charlson Comorbid Index ≥1**	70.0/37.1*	60.9/69.4^#^	63.6/37.8*	85.0/97.0*	73.1/71.5	67.1/52.9^#^
**Comorbid conditions**						
**COPD**	6.6/4.0	14.2/12.3	4.0/2.0	2.5/3.4	3.8/3.3	1.2/2.6
**Congestive heart failure**	11.9/2.6*	8.6/5.5^#^	3.1/0.6^#^	12.5/3.4^#^	12.8/3.7^#^	5.9/2.9
**Cerebrovascular disease**	11.5/3.8*	16.6/21.8^#^	7.6/2.1*	8.8/3.0^#^	10.3/7.0	2.4/4.0
**Diabetes mellitus**	32.6/21.2*	21.2/25.8	15.6/16.4	16.3/21.2	23.1/23.6	44.7/33.8
**End stage renal disease**	7.0/2.8*	6.6/4.1	2.7/2.2	47.5/52.2	17.9/9.1^#^	5.9/2.9
**Cirrhosis**	8.8/2.8*	1.3/1.9	1.8/1.7	1.3/2.4	5.1/0.2*	11.8/2.9^#^
**Cancer**	9.3/4.1*	5.6/7.2	29.3/15.6*	18.8/38.0^#^	2.6/2.6	9.4/1.5*
**Infections other than SST**	51.1/18.0*	78.5/56.6*	44.9/21.2*	62.5/38.4*	50.0/17.8*	56.5/22.8*
**Hospital mortality, %**	14.5/0.6*	20.2/6.2*	8.9/0.5*	17.5/3.4*	23.1/2.8*	28.2/1.5*

^$ ^Digits in cells represent percentages; * *p *< 0.001; ^#^*p *< 0.05; Center/Regional/District: Medical center/Regional hospital/district hospital; COPD: chronic obstructive pulmonary disease; SST: skin/soft tissue.

### Factors associated with ICU admission and hospital mortality for SSTIs

Factors associated with ICU admission and hospital mortality for the six SSTIs are shown in Table 4 and Table 5, respectively. After adjusting for demographics and hospital levels, the presence of several comorbid conditions was associated with ICU admission or hospital mortality, but the results were inconsistent across most common SSTIs. In the same analyses, the presence of non-SST infections was consistently associated with increased risk of ICU admission (adjusted odds ratios [OR] 3.34, 95% confidence interval [CI] 2.91-3.83, for all SSTI patients) and hospital mortality (adjusted OR 5.93, 95% CI 4.57-7.71).

**Table 4:** Multivariate analyses for factors associated with intensive care unit admission (Forward logistic regression model)*

Variables	Other cellulitis or abscess	Decubitus ulcer	Post-operation wound infection	Infection due to vascular device/implant	Gangrene	Necrotizing fasciitis
**Age ≥65 yr**	1.88(1.40-2.52)		2.43(1.75-3.39)			
**Surgical conditions**	5.49(4.04-7.45)	2.46(1.75-3.46)	13.38(7.73-23.17)	2.54(1.40-4.62)		
**Hospital level**						
**Medical center**	1	1				1
**Regional hospital**	0.90(0.65-1.24)	1.54(1.06-2.24)				0.38(0.21-0.69)
**District hospital**	0.56(0.38-0.83)	0.95(0.65-1.40)				0.25(0.10-0.61)
**Comorbid conditions**						
**Congestive heart failure**	5.01(3.09-8.11)	1.89(1.13-3.19)	4.07(1.03-16.18)	4.07(1.50-11.06)	4.33(1.78-10.56)	
**Cerebrovascular disease**	3.25(2.01-5.27)		4.67(2.10-10.36)			
**End stage renal disease**				0.27(0.14-0.53)		
**Cirrhosis**	3.42(2.00-5.85)				35.81(3.77-340.61)	4.28(1.46-12.51)
**Cancer**	1.95(1.17-3.25)		2.61(1.78-3.82)	0.17(0.08-0.38)		5.35(1.42-20.16)
**Infections other than SST**	4.51(3.40-5.99)	3.27(2.39-4.49)	2.86(2.04-4.02)	2.61(1.52-4.50)	5.01(2.96-8.49)	4.02(2.33-6.95)

**P-value for goodness-of-fit**	0.081	0.970	0.375	0.846	0.374	0.542
**Nagelkerke pseudo-R^2^**	0.202	0.107	0.314	0.200	0.179	0.242

*Values represent adjusted odds ratio (95% confidence interval). Factors used for multivariate analyses included age ≥65 year, gender, medical/surgical conditions, hospital levels, comorbid conditions and infections other than skin/soft tissue (SST).

**Table 5:** Multivariate analyses for factors associated with hospital mortality (Forward logistic regression model) *

Variables	Other cellulitis or abscess	Decubitus ulcer	Post-operation wound infection	Infection due to vascular device/implant	Gangrene	Necrotizing fasciitis
**Age ≥65 yr**	2.83(1.66-4.83)		4.02(1.62-9.96)		4.35(1.27-14.87)	
**Surgical conditions**	2.14(1.16-3.94)	0.46(0.24-0.84)				
**Comorbid conditions**						
**Congestive heart failure**			9.66(1.72-54.20)			
**Cerebrovascular disease**		0.45(0.24-0.84)				
**End stage renal disease**	3.05(1.28-7.26)					
**Cirrhosis**	2.55(1.06-6.12)			7.84(1.17-52.31)		8.75(2.56-29.80)
**Cancer**	10.44(5.83-18.68)	3.99(2.35-6.75)	5.65(2.39-13.35)		4.39(1.04-18.40)	24.74(6.09-100.39)
**Infections other than SST**	8.43(4.95-14.36)	1.56(1.02-2.38)	9.04(3.61-22.62)	11.04(3.16-38.50)	4.27(1.96-9.29)	7.28(2.80-18.92)

**P-value for goodness-of-fit**	0.265	0.854	0.045	0.452	0.358	0.667
**Nagelkerke pseudo-R^2^**	0.229	0.072	0.242	0.172	0.156	0.319

*Values represent adjusted odds ratio (95% confidence interval). Factors used for multivariate analyses included age ≥65 year, gender, medical/surgical conditions, hospital levels, comorbid conditions and infections other than skin/soft tissue (SST)

## Discussion

In this study, we found that the proportional distributions of various SSTIs differed between ICU and non-ICU patients due to different incidences and ICU admission rates of these SSTIs. The six most common SSTIs in the ICU accounted for 97.2% of the ICU cases and included "decubitus ulcer", "other cellulitis/abscess", "post-operation wound infection", "necrotizing fasciitis", "infection due to vascular device/implant" and "gangrene". By analyses of the six SSTIs, SSTIs appeared to vary greatly in many aspects including demographics, clinical characteristics and risks of ICU admission and hospital mortality. Despite these, non-SST infections, present in nearly one-third of the patients, were consistently associated with increased risks of ICU admission and hospital mortality.

Compared with Edelsberg's study [5], we found more "often fatal infections" (7.2% vs 1.3%), less "deeper or healthcare-associated infections" (35.9% vs 40.1%) and a similar proportion of "superficial infections" (56.9% vs 58.6%) in the hospitalized patients with SSTIs. However, compared with another study on dermatological conditions in the ICU [8], the ICU patients with SSTIs in our study had less "often fatal" (15.0% vs 59.1%) and "superficial" (23.5% vs 36.8%) infections but much more "deeper or healthcare-associated infections" (61.6% vs 4.0%). Reasons for the discrepant results may be due to differences in patient populations, in the ICU triage policies, in the environmental or geographic characteristics or in the inclusion criteria of SSTIs [8]. For example, more patients with non-surgical conditions (72.3% vs 52.8%) and more types of SSTIs (18 vs 8 types of SSTIs) were included in our study [8]. Besides, the ICU admission rates for patients with "superficial" and "deep or healthcare-associated" infections were higher than other reports (9.0% vs 2.0%-5.8%) [14,15].

The odds of ICU admission were consistently increased in the presence of non-SST infections across the six most common SSTIs. Similar findings were observed on the risks of hospital mortality. This finding is novel but not surprising because the occurrence of ≥2 sites of infection usually indicates a severe, secondary or nosocomial infection and/or a poor host immunity, all of which are associated with a poor outcome [22].

The presence of significant underlying comorbidities may complicate or delay response to treatment of SSTIs, leading to increased risks of treatment failure and mortality [7,14,23-26]. In this study, we found that the presence of several comorbid conditions in certain SSTI patients might increase the risks of ICU admission and hospital mortality. For example, the presence of congestive heart failure predicted ICU admission in most SSTI patients except those with "necrotizing fasciitis". But the effect of congestive heart failure on mortality was significant only in those with "post-operation wound infection". On the other hand, the presence of cancer was the most powerful and common predictor of hospital mortality in most SSTI patients except those with "infection due to vascular device/implant". These findings are generally in agreement with prior reports [14,23-26].

Among factors associated with ICU admission and hospital mortality, several inconsistent or contradictory results were observed. For example, (A) in patients with "decubitus ulcer", the presence of surgical conditions increased the odds of ICU admission, but reduced the risk of death. The reason for this contradictory finding is unclear because the severity of illnesses and the indications of surgery were not available, rendering further analyses impossible. Besides, the presence of unmeasured confounders might also have implications. (B) The presence of cerebrovascular disease in patients with "other cellulitis/abscess" or "post-operation wound infection" increased the odds of ICU admission, whereas its presence reduced the risk of hospital mortality in those with "decubitus ulcer". The latter finding is contrary to the results of one study, which, using national mortality data, has shown that the presence of cerebrovascular disease in patients with decubitus ulcer increases the risk of death (OR 1.4, 95% CI 1.4-1.5) [24]. However, because we only looked at the short-term outcome and the study methods are different, the results may not be comparable. And (C) among patients with "infection due to vascular device/implant", the presence of cancer or ESRD was associated with a lower risk of ICU admission, but not related to hospital mortality. Most of vascular device/implant-related infections arise either by infection of the local wound/puncture/exit site or by contamination of the catheter hub, leading to intraluminal colonization and subsequent systemic infection [27,28]. Although "infections due to vascular device/implant" are a common cause of hospitalization in patients with cancer or ESRD, most of them have local or non-critical infection and do not require intensive care [28,29].

This study has several other limitations. First, the definitions of SSTIs in this study relied on the coding in stead of the clinical, microbiological or pathological criteria, the accuracy of the diagnosis and coding could not be verified. Because only five diagnostic codes were available, some cases with SSTIs could have been missed. Second, the quality of data could not be audited for the purpose of research. Third, we could not differentiate the timing for the occurrences of SSTIs, non-SST infections and ICU admission. Therefore, whether these SST and non-SST infections were community-acquired or healthcare-associated could not be ascertained. Fourth, hospital mortality might be underestimated for unable to verify through linkage of death certificate. Finally, detailed analyses were only performed in the six most common SSTIs because of small number in the others. Despite these, our study is strengthened by the large number of patients retrieved from a nationwide population-based dataset, which can provide an unbiased selection and enhance the generalization.

## Conclusions

In conclusion, different proportional distributions of SSTIs were present in ICU and non-ICU patients. Although SSTIs varied greatly in many aspects, the presence of non-SST infections, which occurred in nearly one-third of the patients, consistently increased the risks of ICU admission and hospital mortality across most common SSTIs.

## Abbreviations

CI: Confidence interval; ESRD: End stage renal disease; ICD-9-CM: International Classification of Diseases, Ninth Revision, Clinical Modification; ICU: Intensive care unit; IQR: Inter-quartile range; NHI: National Health Insurance; NHIRD: National Health Insurance Research Database; NHRI: National Health Research Institute; OR: Odds ratio; SST: Skin/soft tissue; SSTI: Skin and soft tissue infection.

## Competing interests

Shen HN has no conflicts of interest to disclose.

Lu CL has no conflicts of interest to disclose.

## Authors' contributions

HNS designed the study, obtained funding, performed data mining and processing, did statistical analyses, drafted the initial manuscript and revised important content. CLL contributed to the analyses and interpretation of results and the revision for important content. All authors read and approved the final manuscript.

## Pre-publication history

The pre-publication history for this paper can be accessed here:

http://www.biomedcentral.com/1471-2334/10/151/prepub
